# Constituents of Propolis: Chrysin, Caffeic Acid, *p*-Coumaric Acid, and Ferulic Acid Induce PRODH/POX-Dependent Apoptosis in Human Tongue Squamous Cell Carcinoma Cell (CAL-27)

**DOI:** 10.3389/fphar.2018.00336

**Published:** 2018-04-06

**Authors:** Katarzyna Celińska-Janowicz, Ilona Zaręba, Urszula Lazarek, Joanna Teul, Michał Tomczyk, Jerzy Pałka, Wojciech Miltyk

**Affiliations:** ^1^Department of Pharmaceutical Analysis, Faculty of Pharmacy, Medical University of Białystok, Białystok, Poland; ^2^Department of Medicinal Chemistry, Faculty of Pharmacy, Medical University of Białystok, Białystok, Poland; ^3^Department of Pharmacognosy, Faculty of Pharmacy, Medical University of Białystok, Białystok, Poland

**Keywords:** proline, proline oxidase, propolis, flavonoids, phenolic acids, collagen, SCC

## Abstract

Propolis evokes several therapeutic properties, including anticancer activity. These activities are attributed to the action of polyphenols. Previously it has been demonstrated, that one of the most abundant polyphenolic compounds in ethanolic extracts of propolis are chrysin, caffeic acid, *p*-coumaric acid, and ferulic acid. Although their pro-apoptotic activity on human tongue squamous cell carcinoma cells (CAL-27) was established previously, the detailed mechanism of this process remains unclear. Considering the crucial role of proline metabolism and proline dehydrogenase/proline oxidase (PRODH/POX) in the regulation of cancer cell survival/apoptosis, we studied these processes in polyphenol-treated CAL-27 cells. All studied polyphenols evoked anti-proliferative activity, accompanied by increased PRODH/POX, P53, active caspases-3 and -9 expressions and decreased collagen biosynthesis, prolidase activity and proline concentration in CAL-27 cells. These data suggest that polyphenols of propolis induce PRODH/POX-dependent apoptosis through up-regulation of mitochondrial proline degradation and down-regulation of proline utilization for collagen biosynthesis.

## Introduction

Historically, propolis has been a key element of healthcare, and it products are still widely used as an alternative and complementary therapy or often as a primary treatment. Ethnopharmacology proved that propolis has medicinal properties, setting out directions for further research. Studies on the biological activity of propolis conducted in recent years revealed a wide spectrum of its activities. Propolis has many therapeutic properties, including antibacterial, antifungal, antiviral, cytotoxic, antioxidant, anti-inflammatory as well as immunomodulatory activity (Bankova et al., 2014).

The chemical composition of propolis is related to its region of origin, however, some compounds occur in a comparable amount in all propolis samples (Popova et al., 2015, 2017a,b; Graikou et al., 2016). To broadly known propolis components belong among others: diterpenes, lignans, sesquiterpenes, acetophenones (Bankova, 2005), flavonoids and phenolic acid esters (Bankova et al., 2000) which are responsible for numerous of its biological activities (Kubiliene et al., 2015; Turan et al., 2015; Yildirim et al., 2016).

Recently, the interest is focused on anticancer properties of propolis. In cancer cells treated with propolis significant changes in various intracellular amino acids and their metabolites were found, among which proline was proved to significantly affect cell viability. The increase in proline concentration was observed in numerous cancer cell lines. The phenomenon was related to cancer survival (Surazynski et al., 2008a) and poor patient prognosis. However, proline degradation by PRODH/POX was found to induce apoptosis in several cancer cell lines (Sadowska et al., 2013; Kononczuk et al., 2015a). Therefore, compounds that modulate intracellular proline metabolism are of great importance as potential anticancer factors.

An important source of proline is collagen. The final products of collagen degradation in lysosomes are imidodipeptides (containing C-terminal proline) that are further hydrolyzed to amino acids by cytoplasmic imidodipeptidase–prolidase [E.C. 3.4.13.9]. Therefore, prolidase plays an important role in the regulation of proline-dependent functions. One of the regulatory role of prolidase is providing proline for collagen re-synthesis. Alternatively, proline is degraded into pyrroline-5-carboxylic acid (P5C) in mitochondria by PRODH/POX. The conversion generates superoxide anion that may contribute to ROS-dependent induction of apoptosis (Liu et al., 2006; Phang et al., 2008a,b). Several studies established the function of PRODH/POX as a tumor suppressor gene. Therefore, the inhibition of its activity is correlated with a cancer progression. In several cancer cells, as cancer of kidney or cancers of digestive tract, the expression of PRODH/POX is very low or not detected (Phang and Liu, 2012).

Proline can be also synthesized either from ornithine by ornithine aminotransferase (OAT) or from glutamic acid by P5C synthase, then P5C might be converted to proline by P5C reductase (PYCR), leading to increase in proline concentration. These pathways are therefore considered in studied on proline-dependent responses of cancer cells.

The squamous cell carcinoma (SCC) is one of the most frequent cancers of the oral cavity cancer [oral squamous cell carcinoma (OSCC)]. It is characterized as highly invasive with the ability to give early and extensive lymph nodes metastases (Carvalho et al., 2004). The cancer treatment is primarily focused on the resection of a tumor. Afterward, the radiotherapy and the chemotherapy are followed. Up to date, therapeutic protocols do not offer to the patients with advanced SCC, effective therapeutic options. The lack of any advanced alternative pharmacotherapy of SCC is a problem of modern medicine (Pilch and Bouquot, 2005).

Several studies revealed the connection between the overexpression of oral cancer overexpressed 1 protein (ORAOV1) and the occurrence of more aggressive tumors in esophagus (Zhai et al., 2014). Additionally, ORAOV-1-overexpressed cell lines, among others CAL-27 cell line, are marked with a higher intracellular proline concentration and stronger resistance on treatment (Jiang et al., 2008, 2010). It was confirmed that ORAOV1 influences PYCR activity. Hence, the increase in intracellular proline level was observed and consequently the promotion of tumor progression (Togashi et al., 2014). Hence, the effect of chosen polyphenols on proline metabolism may be a relevant issue in the apoptosis induction (Phang et al., 2012).

The purpose of the study was to identify mechanisms of cytotoxic activity of selected propolis components considering their effect on the proline metabolism in CAL-27 cell line. Previous analysis of the commercially available, standardized preparations of propolis allowed to identify a few of the most abundant polyphenols as chrysin, caffeic acid, *p*-coumaric acid, and ferulic acid (Maciejewicz et al., 2001; Markiewicz-Żukowska et al., 2012; Czyżewska et al., 2015, 2016). The study revealed the proapoptotic effect of these compounds on CAL-27 cell line (Czyżewska et al., 2016), however, its molecular mechanism still remains unclear. In this report, we present their effect on the apoptosis in tongue squamous cell carcinoma (CAL-27) and the mechanism of the activity.

## Materials and Methods

### Cell Culture

The studies were performed on tongue squamous cell line (CAL-27). The cell line was purchased in ATCC (CRL-2095 TM, American Type Culture Collection, Manassas, VA, United States). Cell culture was maintained in Dulbecco’s modified Eagle’s medium (Gibco, United States) with 10% fetal bovine serum (FBS) (Gibco, United States) and 50 U/mL penicillin, 50 μg/mL streptomycin (Gibco, United States) and incubated at 37°C in 5% CO_2_.

### Polyphenols Solutions

Chrysin was purchased from Roth (Karlsruhe, Germany), *p*-coumaric acid, caffeic acid, and ferulic acid were purchased from Sigma-Aldrich (Steinheim, Germany). All chemicals and reagents used in this study were of analytical grade (purity ≥ 99%). Solutions were prepared directly before adding to cell cultures in appropriate concentrations.

### Cell Viability Assay

Cytotoxicity effects of studied polyphenols on CAL-27 cells was determined using methyl thiazolyl tetrazolium (MTT) according to modified Carmichael’s method. MTT assay bases on the conversion of yellow tetrazolium bromide MTT solution to the purple formazan derivatives, by the mitochondrial enzyme succinate dehydrogenase, occurred in viable cells. Cells (10^5^/mL) were cultured on 96-well plates to obtain 70% of confluency. Then cells were incubated with studied polyphenols for 24 h. Polyphenolic compounds were added as DMSO solution prepared directly before assay. The final concentration of DMSO did not exceed 0.1% in each well. The tested polyphenols were used at the following concentrations: chrysin – 5, 25, 50, 80 μg/mL; ferulic acid – 50, 100, 150 μg/mL, caffeic acid – 65, 130, 190 μg/mL; and *p*-coumaric acid – 70, 140, 210 μg/mL. The concentrations correspond to the following molar concentrations: chrysin – 0.020, 0.098, 0.216, 0.315 mM; caffeic acid – 0.361, 0.722, 1.055 mM; ferulic acid – 0.257, 0.515, 0.772 mM; and *p*-coumaric acid – 0.426, 0.853, 1.279 mM. Cisplatin (CDDP) (Sigma-Aldrich, Germany) (10 μM) was used as a positive control. The selection of polyphenols concentration was made on the grounds of previously received data (Czyżewska et al., 2016). Then followed the incubation for 24 h, then the liquid was removed and cells were washed with PBS. Afterward, 50 μL of MTT solution (1.0 mg of MTT per 1 mL of PBS) was added to each well and incubated for 1 h. To dissolve formazan derivatives dimethyl sulfoxide was added after MTT solution removing. The absorbance was measured at 570 nm wavelength using an Asys UVN 340 microplate reader (Biogenet, Józefów, Poland). For the treated cells, amount of viable cells was expressed as the percentage of control.

### Proliferation Assay

The proliferation of studied cells was evaluated as [methyl-^3^H]thymidine (Hartman Analytic GmbH, Braunschweig, Germany) incorporation into DNA of cells treated with studied polyphenols. In 24-well tissue culture dishes, cells were plated at 1 × 10^5^ cells/well, using 1 mL of growth medium in each well. Various concentrations of polyphenols were added to the culture wells. Cisplatin (10 μM) was used as a positive control. After incubation for 24 h at 37°C, 0.5 μCi/mL of [methyl-^3^H]thymidine (6.7 Ci/mmol) was added to the wells and cultures were incubated at 37°C for 4 h. Thereafter, the cells were washed with 0.05 M Tris-HCl and with 5% TCA. Then followed cell lysis in 0.1 M NaOH containing 1% sodium dodecyl sulfate (SDS). Radioactivity was measured in Liquid Scintillation Analyzer Tri-Carb 2810 TR (Perkin-Elmer, United States) after adjustment of 2 mL of scintillation liquid (Ultima Gold XR, Perkin-Elmer, Waltham, MA, United States) to each sample.

### Western Immunoblot

To determine the expression of proteins involved in apoptosis process and proline metabolism western immunoblot was performed. Goat monoclonal anti-PRODH/POX antibody (#EB11136) was purchased from EverestBiotech (United Kingdom). Rabbit anti-caspase-3 (#9665), rabbit anti-cleaved-caspase-3 (#9664), rabbit anti-caspase-9 (#9508), and mouse anti-cleaved-caspase-9 (#7237) antibodies were purchased from Cell Signaling (United States). Purified mouse anti-p53 antibody (#554169) was acquired from Becton, Dickinson and Company (B&D) (United States). Monoclonal anti-β-actin antibody produced in mouse was obtained from Sigma-Aldrich (#A2228, United States). Secondary polyclonal anti-goat, anti-rabbit and anti-mouse antibodies labeled with horseradish peroxidase were purchased in Sigma-Aldrich, Corp. (United States).

The Laemmli method was used to perform SDS-PAGE electrophoresis (Laemmli, 1970). The equal amounts of cell supernatants (15 μg of protein) were analyzed. Semi-dry electrotransfer was performed after electrophoresis. For this procedure, Multiphor II Electrophoresis System Multiphor (GE Healthcare Bio-Sciences AB, Sweden) was used. Subsequently, the sequence of incubation with primary and secondary antibodies and were performed. Afterward, membranes were incubated with Amersham ECL Western Blotting Detection Reagent (GE Healthcare Life Science, United Kingdom), and chemiluminescence was analyzed with BioSpectrum Imaging System UVP (UltraViolet Products, Ltd., United Kingdom). The densitometry of bands was quantified by ImageJ software.

### Intracellular Proline Concentration

After 24 h incubation with chosen polyphenols, cells were scratched and suspended in a cold mixture of water and methanol (*v/v* 80:20). After then a cycle of frost and defrost followed. Lastly, the cell solution was sonicated and preserved at -80°C until further analysis. The used method allowed to determine intracellular concentration of proline and to compare treated cells with untreated ones.

The intracellular level of proline was determined using HPLC system (Agilent Technologies, Germany) connected to QTOF mass spectrometry detector (Agilent Technologies, Germany). Electrospray ionization (ESI) was used as an ion source. The analysis was performed on a HILIC column (Luna HILIC, Phenomenex, United States). Samples were injected in a volume of 2 μL. The procedure required maintenance at 40°C. The system was operated in both positive and negative mode at flow rate 1 mL/min with solvent A – water with 10 mM ammonium formate, and solvent B – acetonitrile/water (9:1, *v:v*) with 10 mM ammonium formate. A mobile phase was as follows: 100% B during 1.5 min in isocratic mode, at 1.5 min the gradient mode started from 100% B to 70% B in 5.5 min, then 40% B in 6.0 min, maintained 40% B during 1 min and returned to starting conditions in 0.5 min, keeping the re-equilibration until 10 min. The detector operated in full scan mode. During analyses, reference masses were delivered continuously to obtain accurate mass measurements. Reference masses were at *m/z* 121.0509 (protonated purine) and *m/z* 922.0098 [HP-921]. Compound identification was performed monitoring monoisotopic ions of proline and the internal standard (Proline-d3, #791261, Sigma-Aldrich, United States). The concentration range of proline to which calibration curve was registered was as followed: 6.32–101.12 [μM]. Each of sample was analyzed in triplicates. To further calculations the average value of signals was used. Obtained concentrations were normalized to the internal standard and to the amount of protein in a sample.

### Vitality Assay

Vitality assay describes changes in the intracellular level of (reduced) thiols such as glutathione which exists in two forms: reduced – GSH and – oxidized state (GSSG). The decrease in GSH levels is an early signal of cell death caused either by the direct GSH oxidation promoted by radicals or by the export of GSH through an ATP-dependent plasma membrane transport system. The assay was performed using NucleoCounter^®^ NC-3000^TM^ system (ChemoMetec, Denmark) according to manufacturer’s protocol. Cells incubated with studied concentrations of polyphenols were detached from the plates, washed and stained with Solution 5 (containing three various reagents: VitaBright-48, propidium iodide, and acridine orange). Cisplatin (10 μM) was used as a positive control. Thereafter, the intensity of fluorescence was measured using NucleoCounter^®^ NC-3000^TM^.

### Determination of Prolidase Activity

The activity of prolidase was determined according to the method of Myara et al. (1982). It is colorimetric assay using Chinard’s reagent. To perform the assay cells were detached and scraped off using Cell Lysis Buffer (Cell Signaling, United States). *N*-Benzyloxycarbonyl-L-proline (Cbz-Pro) (Bachem, United States) (5 mM) was used as a positive control. Then samples were sonicated for 3 s × 10 s at 0°C and then centrifuged at 16000 ×*g* for 15 min. Afterward, the cell pellet was discarded. Supernatants were used for protein determination according to Lowry method and evaluation of prolidase activity. The activation of prolidase requires incubation of cell extract supernatant with Mn (II), due to this fact the 100 μL of each sample was mixed with 100 μL of 50 mM HEPES (pH 7.8) containing MnCl_2_ to achieve the final concentration 1 mM of MnCl_2_. Incubation lasts for 24 h at 37°C then the prolidase reaction was initiated by adding 100 μL of the activation mixture which was 94 mM glycyl-proline (Gly-Pro). After adding of Gly-Pro samples were incubated for 1 h at 37°C. The reaction was terminated by addition 1 mL of 0.45 M trichloroacetic acid. Parallel blank tubes contained trichloroacetic acid was added at time “zero.” After the samples centrifugation (10.000 ×*g* for 15 min) the amount of released proline was determined by adding trichloroacetic acid/supernatant to the mixture of glacial acetic acid: Chinard’s reagent. Then followed incubation for 10 min at 90°C. The determination of proline was based on the measurement of absorbance at 515 nm. Prolidase activity was expressed as nanomoles of proline released per minute per milligram of protein.

### Collagen Biosynthesis

Collagen biosynthesis was measured as the incorporation of radioactive proline into proteins. CAL-27 were incubated with various concentrations of polyphenols and with the 5 μCi/mL of 5[^3^H]-proline (Hartman Analytic GmbH, Germany) for 24 h. 2-Metoxyestradiol (MOE) (Sigma-Aldrich, United States) (10 μM) was used as a positive control. The amount of incorporated 5[^3^H]-proline into collagen was detected after its digesting by *Clostridium histolyticum* collagenase (Sigma-Aldrich, United States) according to the method of Peterkofsky and Diegelmann (1971).

### Statistical Analysis

Statistical analysis was conducted using GraphPad PRISM v. 5 (GraphPad Software, Inc., San Diego, CA, United States). For results from all experiments, the mean values for at least six measuring points ± standard error of the mean (SEM) were determined. The statistical significances were calculated using one-way ANOVA with Dunnett’s multiple comparison tests. Differences at *p* < 0.001 were accepted as statistically significant.

## Results

### Studied Polyphenols Significantly Reduce the Viability of CAL-27 Cancer Cells

**FIGURE 1:**
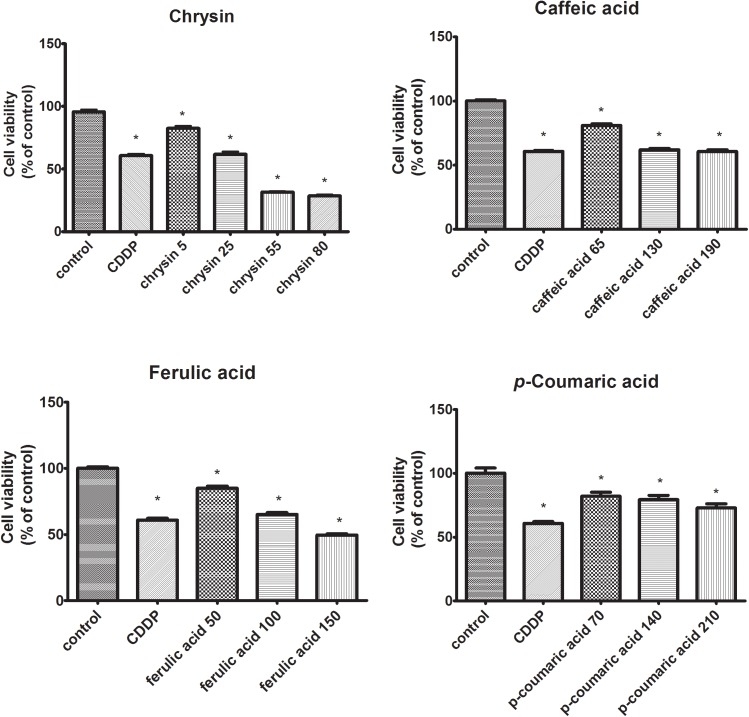
The cytotoxic effects of polyphenolic compounds on CAL-27 cells. The cells were treated with a specified concentration [μg/mL] of respective components for 24 h and cytotoxic effects were determined by MTT assay. Cisplatin (CDDP) (10 μM) was used as a positive control. The values represent mean ± SEM of two independent experiments conducted six times (*n* = 12). The stars indicate that value were significantly different (^∗^*p* < 0.001) compared with control.

The influence of chosen polyphenols (chrysin, caffeic acid, *p*-coumaric acid, ferulic acid) on the viability of human tongue squamous cell carcinoma cell line (CAL-27) was determined with MTT assay. The studied polyphenols ability to induce the cytotoxic effect in CAL-27 was observed for all polyphenolic compounds (**Figure 1**). The use of chrysin resulted in a reduction of cell survival to 82, 62, 32, and 29%. In the case of caffeic acid, decreased CAL-27 cells survival to 81, 62, and 61% was observed. The use of ferulic acid decreased cell survival to 85, 65, and 50% compared to controls whereas in the case of *p*-coumaric acid, the viability of CAL-27 cells was reduced to 82, 80, and 73% compared to controls (100%). To evaluate whether polyphenols cytotoxicity was a result of their influence on cell DNA biosynthesis the capability of intracellular thymidine incorporation was assessed.

### The Inhibition of DNA Biosynthesis After Polyphenols Application

**FIGURE 2:**
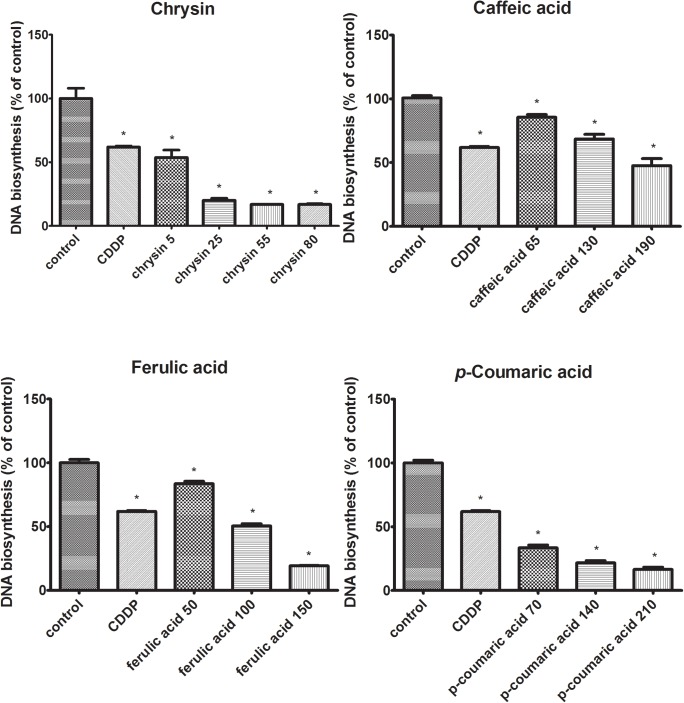
Decreasing effects of polyphenolic compounds on DNA biosynthesis in CAL-27 cells. The cells were incubated with specified concentration [μg/mL] of respective polyphenols for 24 h and [^3^H]-thymidine incorporation into CAL-27 was measured in scintillation counter. Cisplatin (10 μM) was used as a positive control. The values represent mean ± SEM of two independent experiments conducted three times (*n* = 6). The stars indicate that value were significantly different (^∗^*p* < 0.001) compared with control.

The effects of chrysin, caffeic acid, *p*-coumaric acid, ferulic acid on DNA biosynthesis in CAL-27 cells were evaluated as [^3^H]-thymidine incorporation into viable cells. The downregulation of DNA biosynthesis in CAL-27 cells was observed for all polyphenolic compounds (**Figure 2**). The obtained results show that the addition of chrysin resulted in a reduction in DNA biosynthesis by 46, 80, 83, and 83% whereas the application of caffeic acid leads to a reduction of DNA biosynthesis by 14, 31, and 52%. Moreover, the similar situation was observed after adding ferulic acid where the DNA biosynthesis was reduced by 16, 46, 53, and 71%. In the case of using *p*-coumaric acid, DNA biosynthesis was also reduced by 67, 78, and 83%. These findings of indicate that all polyphenols are inhibitors of DNA biosynthesis in CAL-27 SCC cells, and the intensity of this process depends on the concentration of the compound used.

To evaluate whether chosen compounds lead to the cell death through the apoptosis pathway involved proline metabolism regulation the western immunoblot assay was performed. For further analysis polyphenols were used at the lowest doses that contributed to noticeable cytotoxic effects. Applied concentrations were as follows: chrysin – 5 μg/mL, caffeic acid – 65 μg/mL, ferulic acid – 50 μg/mL, and *p*-coumaric acid – 70 μg/mL.

### Selected Polyphenols Activate p53, PRODH/POX, Caspase 9 and 3 in CAL-27 Cell Line

**FIGURE 3:**
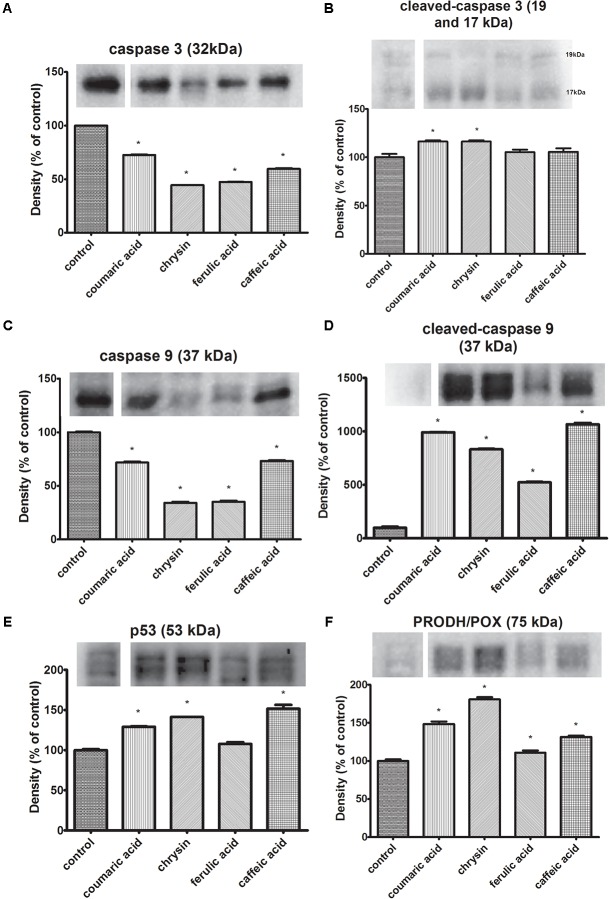
Expression of un-cleaved caspase-3 **(A)**, cleaved-caspase-3 **(B)**, un-cleaved caspase-9 **(C)**, cleaved-caspase-9 **(D)**, PRODH/POX **(F)** and p53 **(E)**, in CAL-27 cells and the effect of: *p*-coumaric acid, chrysin, ferulic acid, and caffeic acid on the process. β-Actin was used as a control (data contained in Supplementary Presentation 1). The values represent mean ± SEM of six pooled cell homogenates extracts from three independent experiments (*n* = 18). The stars indicate which values were significantly different (^∗^*p* < 0.001) compared with control.

The study was conducted applying Western Immunoblot method described in chapter 2.5. CAL-27 cells were subjected to 24 h exposure to selected concentrations of polyphenols: chrysin (5 μg/mL), caffeic acid (65 μg/mL), ferulic acid (50 μg/mL), and *p*-coumaric acid (70 μg/mL). To evaluate the course of apoptosis process the caspases expression were assessed. The findings showed the reduction of expression of total caspase 3 in CAL-27 cells incubated with all of the tested polyphenols. The largest reduction in the expression of the total caspase 3 form compared to the control was observed in cells incubated with chrysin (5 μg/mL) and ferulic acid (50 μg/mL) (**Figure 3A**). Additionally, the obtained data showed reduced expression of total caspase 9 in CAL-27 cells incubated with all polyphenols tested. The most significant reduction in the expression of the total caspase 9 form was observed in cells incubated with chrysin (5 μg/mL) and ferulic acid (50 μg/mL) (**Figure 3C**). Moreover, obtained results revealed an increase in cleaved caspases 9 and 3 expressions in CAL-27 cells after incubation with each of used polyphenols (**Figure 3**). The highest increase in expression of active caspase 9 compared to control was observed in CAL-27 cells exposed to caffeic acid (65 μg/mL) and *p*-coumaric acid (70 μg/mL) (**Figure 3D**). Furthermore, the data revealed increased expression of the active form of caspase 3 in CAL-27 cells incubated with each of the polyphenols tested. The most noticeable increase in expression of active caspase 3 in relation to control was observed in the case of CAL-27 cells exposed to chrysin (5 μg/mL) and *p*-coumaric acid (70 μg/mL) (**Figure 3B**). The following results confirm the pro-apoptotic effect of the polyphenols. These compounds stimulate the expression of the active forms of the initiator caspase 9 involved in the activation of the intrinsic pathway of apoptosis and the executive caspase 3. The initiation of apoptosis through the activation of the p53 protein is accompanied by the induction of proline oxidase (PRODH/POX) expression. It is a mitochondrial enzyme that catalyzes the conversion of proline to pyrroline-5-carboxylate while transferring free electrons to cytochrome c resulting in apoptosis. Therefore, to further examination whether observed apoptosis involved the changes in PRODH/POX activity, the western immunoblot for PRODH/POX and p53 (the most potent inducer of this protein expression) was conducted. Received outcomes demonstrated the increase in PRODH/POX expression in all studied cases (**Figure 3F**). The obtained results coincide with the p53 expression that was increased in all samples. Nonetheless, the lowest increase was observed in CAL-27 cells previously incubated with ferulic acid (**Figures 3E,F**).

### The Proapoptotic Effect of Chrysin, Caffeic Acid, *p*-Coumaric Acid, and Ferulic Acid

**FIGURE 4:**
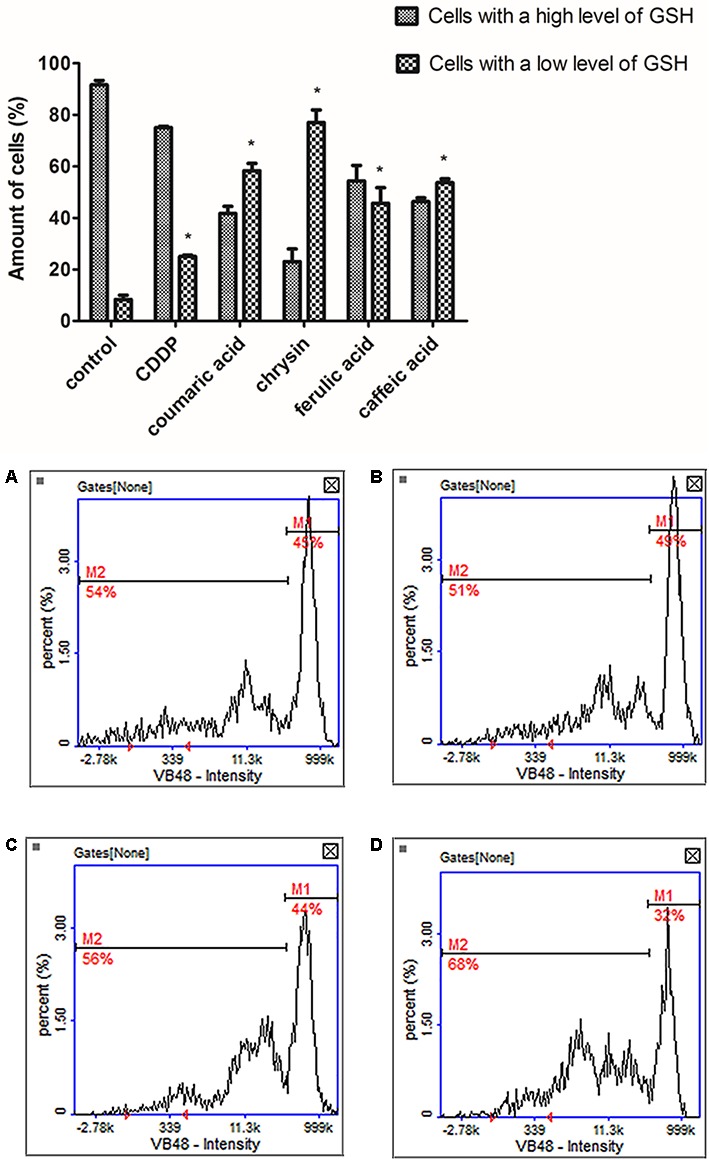
The analysis of cellular reduced glutathione (GSH) levels in CAL-27 cells after 24 h exposure to tested polyphenols in following concentrations: **(C)** chrysin – 5 μg/mL, **(B)** caffeic acid – 65 μg/mL, **(A)** ferulic acid – 50 μg/mL, and **(D)** *p*-coumaric acid – 70 μg/mL. Cisplatin (10 μM) was used as a positive control. M1 represents the cells with a high GSH levels (high vitality); M2 represents the cells with low GSH levels (low vitality). The graphs present mean values ± SEM from three experiments done in duplicates. The asterisks determine values significantly different from control samples (^∗^*p* < 0.001).

To confirm induction of apoptosis by the polyphenols the vitality assay was performed. The cells were subjected to 24 h exposure to tested polyphenols in the following concentrations: chrysin (5 μg/mL), caffeic acid (65 μg/mL), ferulic acid (50 μg/mL), and *p*-coumaric acid (70 μg/mL). The obtained outcomes revealed a significant decrease in cellular GSH concentration in treated cells compared to untreated control (**Figure 4**). The most noticeable increase in the cell population with the low level of GSH was observed in CAL-27 cells treated with chrysin. The loss of GSH is broadly known to be directly related to the apoptosis progression and the assay confirmed our data received from MTT assay.

The performed assays confirmed the apoptosis induction in treated cells. To estimate whether chosen compounds leads to apoptosis through the disruption of proline metabolism, further studies were conducted.

### Chrysin, Caffeic Acid, *p*-Coumaric Acid, and Ferulic Acid Affect the Cellular Metabolism of Proline

**FIGURE 5:**
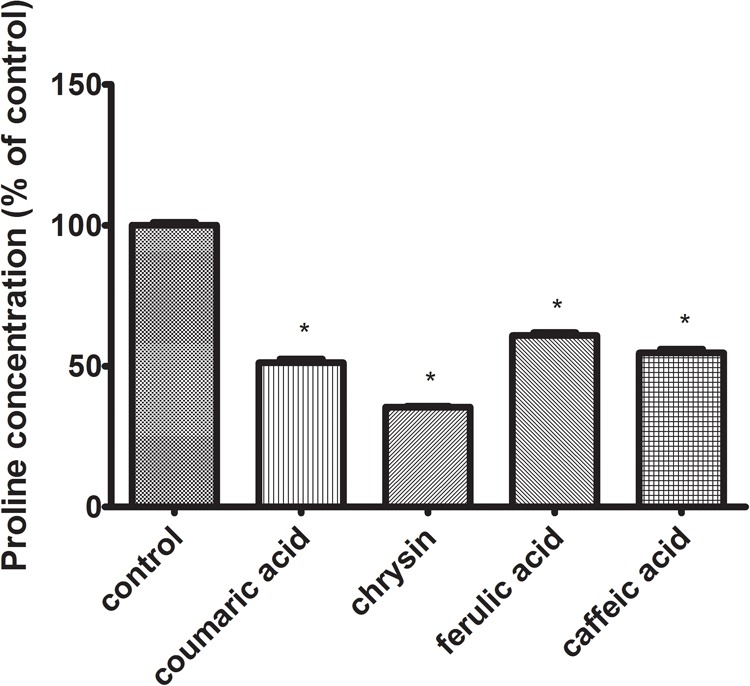
The effect of 24 h exposure to *p*-coumaric acid (70 μg/mL), chrysin (5 μg/mL), ferulic acid (50 μg/mL), and caffeic acid (65 μg/mL) on intracellular proline concentration in CAL-27 cell culture. The mean values ± SEM from three experiments done in duplicates are presented (*n* = 6). The stars indicate which values were significantly different (^∗^*p* < 0.001) compared with control.

Proline transformations affect the regulation of cellular metabolism and apoptosis. The effect of proline on the metabolism of tumor cells seems to be particularly important, where a significant increase in the concentration of this amino acid is observed in comparison with normal cells. The high concentration of intracellular proline is associated with an enhanced ability of tumor cells to metastasize. The performed studies showed the pro-apoptotic activity of all tested polyphenols. The next stage of the study was to determine the effect of the tested polyphenols on the proline level in CAL-27 SCC cells. For evaluation, if discerned changes might induce the adjustments in proline metabolism and hence in its intracellular concentration, the HPLC-QTOF system was used. Data acquired after chromatographic analysis displayed a significant decrease in the intracellular proline concentration in case of CAL-27 cells treated with all studied polyphenols (**Figure 5**).

### The Decline of Collagen Biosynthesis in the Cells Treated With Polyphenols

**FIGURE 6:**
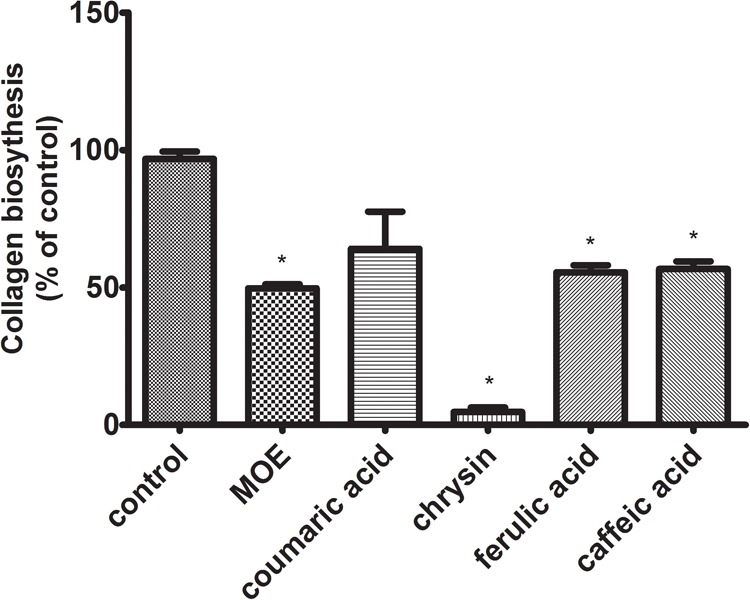
Collagen biosynthesis measured as 5[^3^H]-proline incorporation into proteins susceptible to the action of bacterial collagenase in CAL-27 cell culture incubated for 24 h with polyphenols in following concentrations: chrysin – 5 μg/mL, caffeic acid – 65 μg/mL, ferulic acid – 50 μg/mL, and *p*-coumaric acid – 70 μg/mL. 2-Metoxyestradiol (MOE) (10 μM) was used as a positive control. The values represent mean ± SEM of two independent experiments conducted three times (*n* = 6). The stars indicate values significantly different from control (^∗^*p* < 0.001).

Collagen is a most abundant protein in the extracellular matrix (ECM), where in addition to supporting functions, it participates in the modulation of cellular signals by interacting with integrin receptors. It affects a number of cell processes, including regulation of gene expression, growth, differentiation, and metabolism of cells. It has been observed that in cancer cells, this mechanism is disrupted due to the increased activity of metalloproteinases (MMPs), contributing to the increased degradation of collagen. The biosynthesis level of this protein may reflect changes in metabolic processes in living cells. Since collagen is reported to be the essential source of proline further experiment was designed to assess the possible disorders of collagen biosynthesis after exposure to studied polyphenols. The assay was performed according to the Peterkofsky’s method. Obtained data revealed the decline of collagen biosynthesis in the cells treated with all tested compounds (**Figure 6**). The reduction of collagen biosynthesis by 36% – *p*-coumaric acid, 95% – chrysin, 44% – ferulic acid, and 43% – caffeic acid, compared to control (100%) was observed. The most noticeable decrease in collagen biosynthesis was observed in the cells incubated with chrysin.

### Inhibition of Prolidase Activity by Tested Polyphenols

**FIGURE 7:**
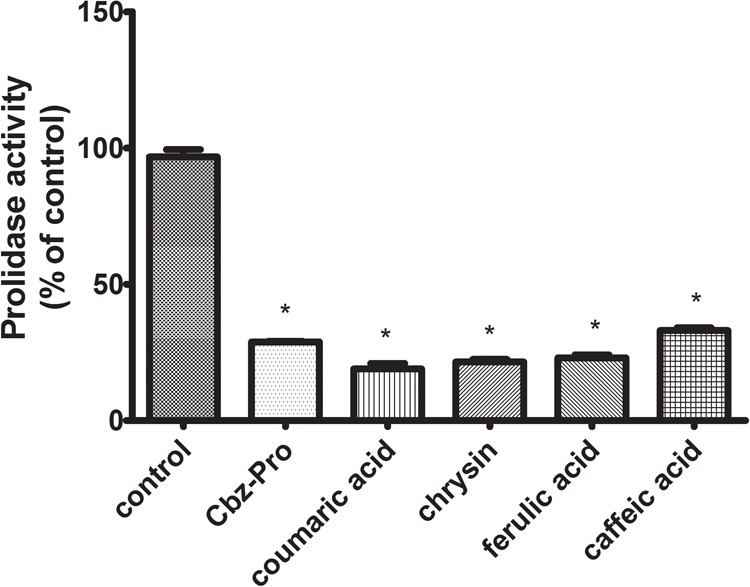
Prolidase activity in CAL-27 cells treated for 24 h with chosen polyphenols (chrysin – 5 μg/mL, caffeic acid – 65 μg/mL, ferulic acid – 50 μg/mL, and *p*-coumaric acid – 70 μg/mL). *N*-Benzyloxycarbonyl-L-proline (Cbz-Pro) (5 mM) was used as a positive control. The mean values ± SEM from three experiments done in duplicates (*n* = 6) are presented. Asterisks indicate differences between studied cells compared to control untreated cells at ^∗^*p* < 0.001.

Prolidase [E.C. 3.4.13.9] is a key enzyme involved in the process of collagen biosynthesis. This enzyme is involved in the breakdown of imidopeptides that are degradation products of collagen fibers. In the last stage of collagen degradation, prolidase catalyzes the reaction of the release of proline and hydroxyproline imidopeptides. The released proline can then be included in the collagen biosynthesis or other metabolic pathways, e.g., related to the process of apoptosis or the neoangiogenesis process. Since proline is the major constituent of collagen (it represents the main source of the aminoacids), their metabolism is directly connected. Due to this fact, prolidase plays a crucial role in the recycling of proline for collagen biosynthesis. In CAL-27 cells incubated with test polyphenols (chrysin – 5 μg/mL, caffeic acid – 65 μg/mL, ferulic acid – 50 μg/mL, *p*-coumaric acid – 70 μg/mL), a reduction in the activity of prolidase was observed, by 81% – *p*-coumaric acid, 78% – chrysin, 77% – ferulic acid, 66% – caffeic acid in relation to the control (**Figure 7**).

## Discussion

The SCC is one of the most frequent cancers of the oral cavity. It is associated with high invasiveness and ability to induce early and extensive lymph nodes metastases (Carvalho et al., 2004). The lack of advanced pharmacotherapy of SCC requires searching for new effective pharmacological strategies (Pilch and Bouquot, 2005). In this study, we found that selected polyphenols of propolis induced apoptosis in CAL-27 cells through up-regulation of proline metabolism.

Recent studies revealed the enhanced progression of the SCC due to up-regulation of proline level induced by amplified genes expression of ORAOV1 and P5C-reductase (Togashi et al., 2014). Therefore, we considered proline metabolism as a target for potential treatment of SCC. In fact, proline bearing reducing potential is considered as a stress molecule. Its metabolism plays an important role in maintaining energetic and redox balance in cells. Free cytoplasmic proline may up-regulate transcriptional activity of HIF-1α that is considered as pro-survival and inflammatory transcription factor since it induces expression of COX-2, VEGF, TNF-α, IL-1, NF-κB (Surazynski et al., 2008a).

Proline could be removed from the cytoplasm by incorporation into collagen. Collagen biosynthesis may serve as a sink for proline. Up-regulation of collagen biosynthesis can remove reducing potential, but it may contribute to tissue cirrhosis usually accompanying prolonged inflammation (Priest and Davies, 1969; Davis and Cowie, 1990). Interesting is a fact of significant alterations in collagen content in SCC tissue. Agarwal and Ballabh indicated a definite alteration in the distribution of collagen type IV with its significant loss in poorly-differentiated SCC tissue (Agarwal and Ballabh, 2013), related to increased metastasis. On the other hand, proline is generated from imidodipeptides (derived from collagen degradation products) by imidopeptidase – prolidase. Therefore, the enzyme activity represents an important factor in the regulation of intracellular proline concentration. However, other mechanisms that contribute to generation as well as, utilization of intracellular proline are as well-important.

A critical pathway of proline utilization is mitochondrial degradation by proline oxidase – PRODH/POX. During this process ROS and/or ATP are generated. PRODH/POX is widely distributed in living organisms and is responsible for a number of regulatory processes such as redox homeostasis, osmotic adaptation, cell signaling, and oxidative stress. Recent data provided evidence that enzyme plays a crucial role in inhibition of carcinogenesis and tumor growth. It may induce apoptosis in both intrinsic and extrinsic ways in cancer cells (Zareba and Palka, 2016). Unfortunately, the enzyme is often down-regulated in cancer cells leading to inhibited apoptosis (Kononczuk et al., 2015a). Our results indicated almost undetectable expression of PRODH/POX in CAL-27 cells. Therefore, we analyzed propolis compounds as factors inducing PRODH/POX expression and decreasing proline level. We suggest that polyphenols contained in propolis induced the enzyme activity, leading to induction of apoptosis in CAL-27 cells.

We found that studied polyphenols decreased in a dose-dependent manner the viability of CAL-27 cells. Moreover, the treatment inhibited DNA biosynthesis in these cells. The decrease in cell number was related to induction of apoptosis. We observed a significant increase in expression of active/cleaved caspases -3 and -9 and increase in the amount of the cells with low level of reduced glutathione (GSH) in polyphenol-treated CAL-27 cells. Considering the important role of PRODH/POX in apoptosis (Phang, 2017), its expression was evaluated. The polyphenols induced expression of PRODH/POX in CAL-27 cells. Cell growth arrest related to apoptosis is induced by p53. The transcription factor is the most potent inducer of PRODH/POX expression (Nagano et al., 2017). In fact, p53 expression was also induced by studied polyphenols in SCC cells.

PRODH/POX-dependent utilization of proline in polyphenol-treated CAL-27 cells was confirmed by LC–MS analysis of proline concentration. Since proline could be utilized in collagen, the biosynthesis of this protein was determined. All tested compounds significantly inhibited the process. It suggests that observed decrease in proline concentration was related to its metabolism by PRODH/POX. The decrease of collagen biosynthesis in polyphenol-treated CAL-27 cells may result from inhibition of prolidase by polyphenols. The enzyme plays an important role in collagen biosynthesis (Palka and Phang, 1997; Surazynski et al., 2008b; Kononczuk et al., 2015a). We observed inhibition of prolidase activity in the cells treated with studied compounds (however to various degree). We suggest, that decrease in proline level by propolis compounds result from proline degradation in mitochondria by induced PRODH/POX and not by utilization of this amino acid for collagen biosynthesis. The significant role of PRODH/POX in apoptosis induction we described previously (Kononczuk et al., 2015b). Stimulation of PRODH/POX expression by Eptifibatide, αIIbβ3-integrin inhibitor, lead to strong proapoptotic effect in MCF-7 breast cancer cells.

The present study provides for the first time evidence that compounds of propolis (chrysin, caffeic acid, *p*-coumaric acid, and ferulic acid, of which the most potential effect on CAL-27 cells growth inhibition was attributed to chrysin) contribute to the induction of PRODH/POX-dependent apoptosis in CAL-27 cells. Therefore, PRODH/POX may represent a novel target for the propolis-induced anticancer activity in SCC cells.

## Author Contributions

KC-J, UL, and WM: conceived and designed the experiments. KC-J, IZ, and JT: performed the experiments. KC-J, JT, JP, and WM: analyzed the data. KC-J, IZ, JT, UL, and MT: contributed reagents/materials/analysis tools. KC-J, MT, and WM: wrote the paper.

## Conflict of Interest Statement

The authors declare that the research was conducted in the absence of any commercial or financial relationships that could be construed as a potential conflict of interest.
