# Supplementary material for: Constituents of Propolis: Chrysin, Caffeic Acid, p-Coumaric Acid, and Ferulic Acid Induce PRODH/POX-Dependent Apoptosis in Human Tongue Squamous Cell Carcinoma Cell (CAL-27)
Source: Front Pharmacol. 2018 Apr 6;9:336. doi: 10.3389/fphar.2018.00336 (PMC5897514; doi:10.3389/fphar.2018.00336)
Supplement: Supplementary file 1 [file Presentation_1.PDF]

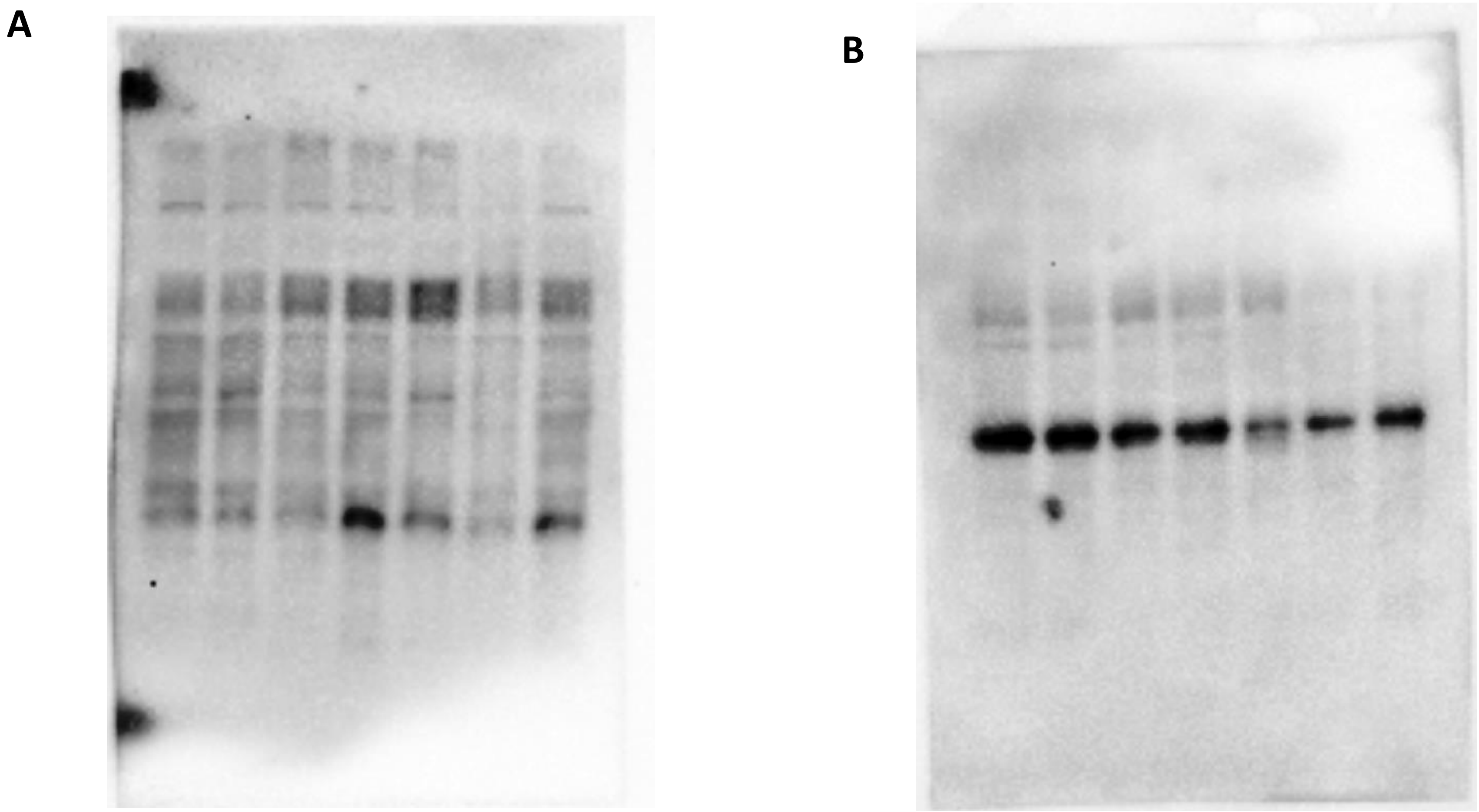

Both photos present results from western immunoblot. Fig.A shows the expression of PRODH/POX protein. Fig.B shows the expression of caspase 3. The first band from the left presents: (Fig.A) PRODH/POX and (Fig.B) caspase 3 expression, in the untreated cells. The second and the third band from the left represents the changes in the expression of these proteins after applying other compounds whose results are planned for publication in a separate publication. The remaining bands show the results for cells treated with: *p*-coumaric acid, chrysin, ferulic acid and caffeic acid, respectively.

A

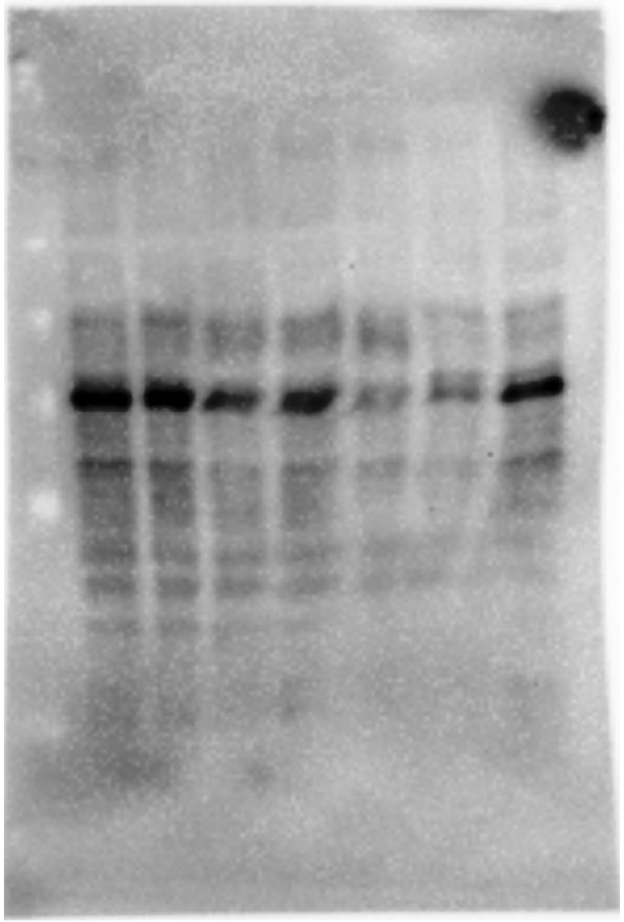

B

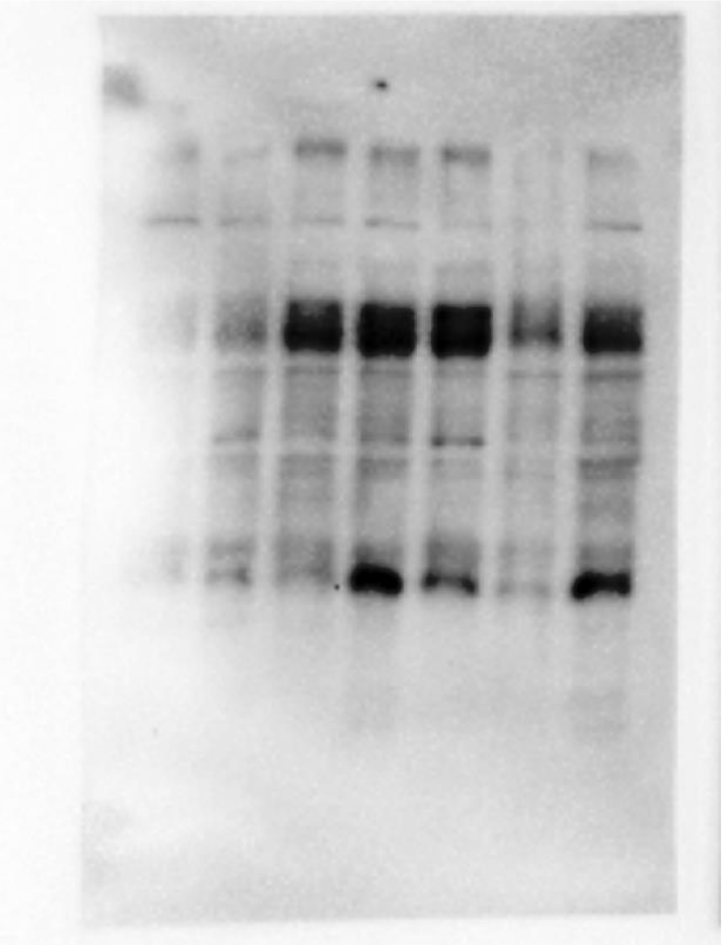

Both photos present results from western immunoblot. Fig.A shows the expression of caspase 9. Fig.B shows the expression of cleaved-caspase 9. The first band from the left presents: (Fig.A) caspase 9 and (Fig.B) cleaved-caspase 9 expression, in the untreated cells. The second and the third band from the left represents the changes in the expression of these proteins after applying other compounds whose results are planned for publication in a separate publication. The remaining bands show the results for cells treated with: *p*-coumaric acid, chrysin, ferulic acid and caffeic acid, respectively.

A

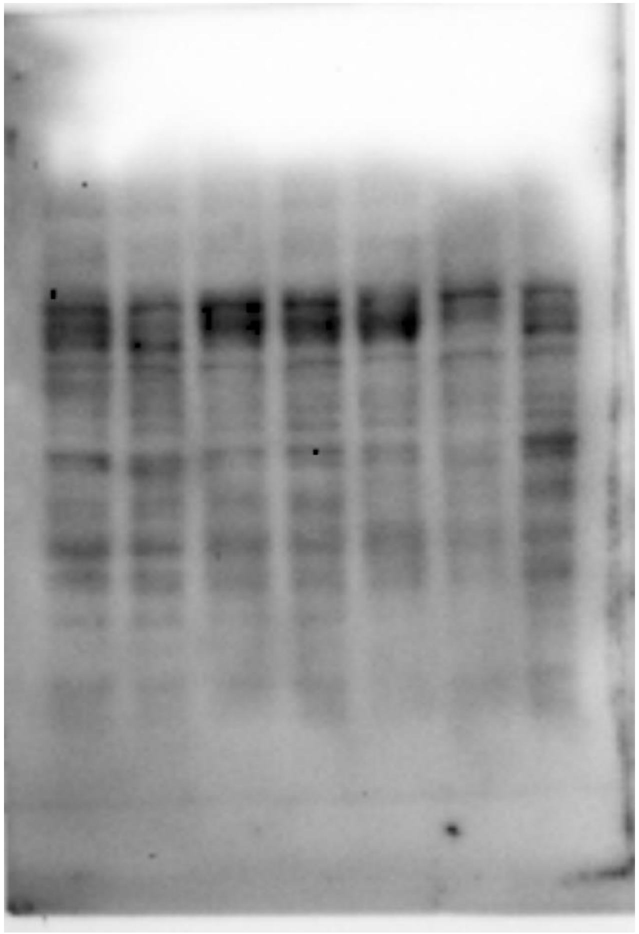

B

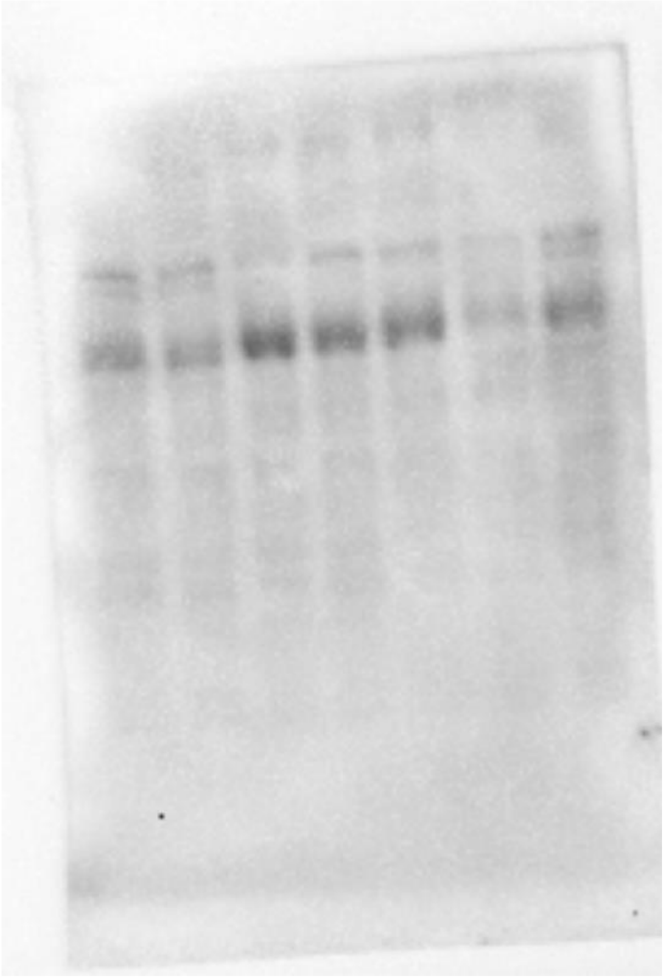

Both photos present results from western immunoblot. Fig.A shows the expression of P53 protein. Fig.B shows the expression of cleaved-caspase 3. The first band from the right presents: (Fig.A) P53 and (Fig.B) cleaved-caspase 3 expression, in the untreated cells. The second and the third band from the right represents the changes in the expression of these proteins after applying other compounds whose results are planned for publication in a separate publication. The remaining bands show the results for cells treated with: *p*-coumaric acid, chrysin, ferulic acid and caffeic acid, respectively.

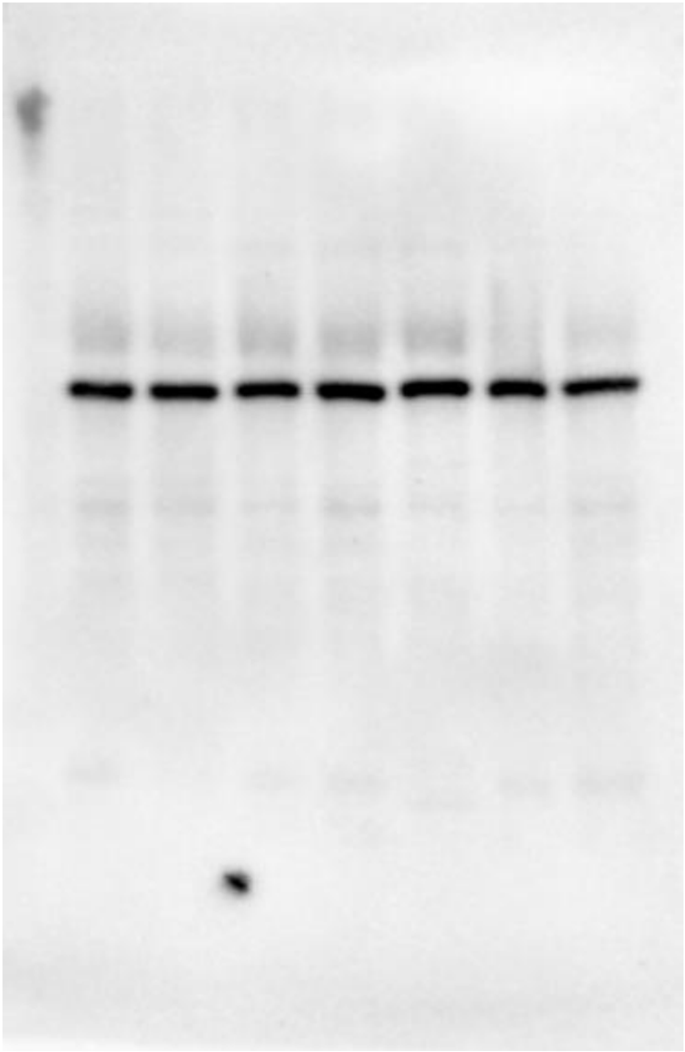

The figure shows the expression of β-actin protein. The first band from the left presents expression in the untreated cells. The second and the third band from the left represents the changes in the expression of these proteins after applying other compounds whose results are planned for publication in a separate publication. The remaining bands show the results for cells treated with: *p*-coumaric acid, chrysin, ferulic acid and caffeic acid, respectively.
